# Post‐ischemia and reperfusion kidney injury is mitigated in a novel complement 5 knockout rat

**DOI:** 10.14814/phy2.70576

**Published:** 2025-11-05

**Authors:** Madison McGraw, Amod Sharma, Dinesh Bhattarai, Neelam Joshi, Neriman Gokden, Seong‐Ok Lee, Gopinath Venugopal, Nirmala Parajuli

**Affiliations:** ^1^ Department of Pharmacology & Toxicology University of Arkansas for Medical Sciences Little Rock Arkansas USA; ^2^ Department of Pathology University of Arkansas for Medical Sciences Little Rock Arkansas USA

**Keywords:** C5 knockout, ischemia, kidney injury, reperfusion

## Abstract

Ischemia‐reperfusion injury (IRI) is the central contributing factor to acute kidney injury (AKI). Kidney tissue that becomes necrotic during this process releases a variety of pro‐inflammatory factors, driving activation of the complement cascade. Complement 5 (C5), in particular, has become an important therapeutic target, yet pharmacologic targeting does not achieve complete inhibition nor target all variants of this abundant protein. Here, we have generated and characterized a novel rat model of CRISPR/Cas9‐mediated global C5 deletion (C5^−/−^). C5^−/−^ rats displayed no differences in growth, blood chemistry, or kidney morphology/function from wild‐type (C5^+/+^) counterparts at baseline. Subsequently, we compared C5^−/−^ rats to C5^+/+^ littermates in a renal IRI model to assess differences in the post‐injury response. Compared to C5^+/+^, C5^−/−^ rats displayed significantly improved kidney injury/function as well as the attenuation of the apoptotic pathway post‐IRI. The circulating immune cell composition was affected by C5^−/−^ post‐injury, with significantly increased NK cells, B cells, and CD8+ T‐cells compared to C5^+/+^, indicating altered inflammatory signaling. Similarly, renal sections showed a reduced level of immune cell infiltration, including macrophages and neutrophils. Collectively, these results demonstrate the generation of an effective rodent model of global C5 deletion and the role of C5 as an injury‐promoting molecule during kidney IRI.

## 1 INTRODUCTION

End‐stage kidney disease (ESKD) is currently the 9th leading cause of death in the United States, with few treatment options available for patients (Hashmi et al., 2024). Dialysis, or manual blood filtering, is a cost‐intensive process that reduces a patient's quality of life and provides only a temporary solution to kidney failure (Lim et al., 2022; Strohmaier et al., 2022). Most patients suffering from ESKD rely upon the success of kidney replacement therapy, but transplant failure introduces sunk costs as well as additional risk for the patient. All transplanted kidneys experience some degree of ischemia during the transplantation process, with the seven out of 10 kidneys that are sourced from deceased donors most egregiously affected (Debout et al., 2015; Kayler et al., 2011; Quiroga et al., 2006). After transplantation, reperfusion of the kidney tissue paradoxically causes further damage, a phenomenon termed ischemia‐reperfusion injury (IRI). IRI has been linked to the activation of the innate immune system, which in turn drives the pro‐inflammatory pathology associated with transplant failure (Arslan et al., 2011; Patel & Eggener, 2011).

The complement system, a key facet of innate immunity that circulates in the serum, displays diverse functions and even more varied pathologies. In addition to known complement‐mediated diseases such as C3 glomerulopathy or paroxysmal nocturnal hemoglobinuria, excessive complement activation is well‐documented in autoimmune disorders, Alzheimer's/neurodegenerative disease, and transplant failure (Dalakas et al., 2020; Ishigooka et al., 2022; Maillard et al., 2015; Spasovski et al., 2022; Terényi et al., 2009). Activation of the cascade in response to pathogens or an alternative pathway triggers the cleavage of C3 into C3a and C3b, from which the C5 convertase is formed, thus beginning the terminal complement pathway. The C5 convertase cleaves C5 into C5a and C5b, two products with well‐defined and separate roles. C5b drives the complement cascade to its endpoint in forming the membrane attack complex (MAC), which attaches to cell surfaces and drives lysis during IRI in the kidney (Zhou et al., 2000). Conversely, the C5a anaphylatoxin propagates potent inflammatory signals via its receptor interactions and promotes immune cell infiltration to the injured kidney post‐IRI (Khanal, 2022; Peng et al., 2012). Blocking the binding of C5a to its receptor, C5aR, via a small molecule antagonist has previously been associated with beneficial outcomes in renal IRI (Arumugam et al., 2003). The C5a‐C5aR interaction most famously occurs on the surface of immune cells to stimulate chemotaxis, but recent work has emerged supporting an intracellular signaling role (Arbore et al., 2017; Nauser & Sacks, 2022; Rahman et al., 2021). Thus, the C5 component of complement functions as a key lynchpin that not only mediates the terminal effects of the cascade but also carries out multiple independent roles across a variety of signaling pathways.

The potential of therapeutically targeting C5 beyond traditional complement diseases is being recognized in the form of several C5 or C5aR‐inhibiting pharmacotherapies currently undergoing clinical trials (Badri et al., 2021; Barratt et al., 2024; Jayne et al., 2017; Lee et al., 2019; Nishimura et al., 2014). However, pharmacologic inhibition or depletion of complement components is often inconsistent and muddles attempts to define the role(s) of complement proteins in vivo. This is because complement proteins such as C5 circulate in abundance in the serum and are swiftly regenerated after depletion. While the primary source of C5 synthesis is the liver, cells in various tissues have been reported to synthesize complement (Arbore et al., 2017). In the kidney, intracellular C5 has been observed predominantly in endothelial and epithelial cell types (Arbore et al., 2017; Khanal, 2022). Additionally, there are structural variants of C5 that may not be bound by therapeutics, as noted in clinical trials with eculizumab, a monoclonal C5 inhibitor (Nishimura et al., 2014). Thus, improved models are needed to illuminate the role of C5 in various pathologies.

Genetic knockout models offer a valuable and indispensable tool in studies that seek to examine the role of complement components in vivo. Genome‐wide deletion ensures a complete reduction of both circulating and intracellular complement, making in‐depth mechanistic studies involving complement proteins possible. This has already been recognized via murine models, such as the C5^−/−^, C5ar1^−/−^, and C5aR2^−/−^ models which have been developed in the mouse (Frank, 1995; Kovtun et al., 2017; Kusakabe et al., 2020; van den Bos et al., 2020). However, the disadvantages of murine models are well‐known: (1) mice do not mimic human physiology as accurately as larger animal models, (2) mice are often not ideal for behavioral work, and (3) the small body size of the mouse increases the difficulty of certain microsurgical procedures, such as rodent organ transplantation. Here, we report the generation and characterization of a novel global C5^−/−^ model utilizing CRISPR‐Cas9 genome editing in the rat. We observed a healthy phenotype in the C5^−/−^ rats similar to C5^+/+^ littermates at baseline. However, the application of acute kidney injury via a surgical model of IRI revealed significantly improved kidney function and morphology in C5^−/−^ rats. These results reinforce the importance of C5 as a therapeutic target in renal IRI, and the novel C5^−/−^ rat model generated in this study will provide an invaluable tool for investigating the role of C5 and its cleaved subunits in complement‐related pathologies, including IRI.

## 2 RESULTS

### 2.1 Generation of a novel C5 knockout rat model

**FIGURE 1:**
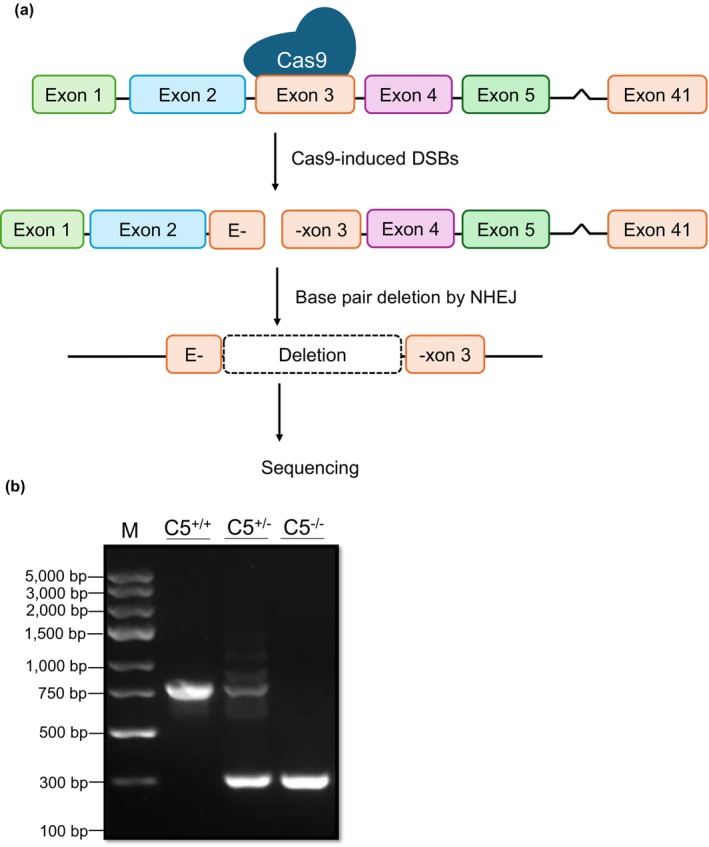
Generation of the C5^−/−^ model. (a) Schematic of CRISPR/Cas9‐mediated C5 gene deletion in rat. CRISPR/Cas9‐mediated knockout of C5 was performed by inducing double‐strand breaks in C5‐201 exon 3 in fertilized rat eggs. Base pair deletion by nonhomologous end‐joining (NHEJ) resulted in mRNA with premature termination codons. (b) Crude DNA extracts prepared from tail snip biopsies of wild‐type (C5^+/+^), heterozygous (C5^+/−^), and homozygous (C5^−/−^) rats were amplified with C5 PCR primer and the PCR product was resolved on an agarose gel (1.5%) followed by visualization using iBright 1500. The 2 kbp DNA ladder was used as a reference marker (M). Representative micrograph shows DNA gel image with 800 bp wild‐type (C5^+/+^) allele and the C5 deletion allele at 334 bp.

To understand the physiological role of C5 in rats, CRISPR‐Cas9 technology was used to generate Lewis background rats carrying a C5 exon 3 deletion allele (50% knockout, C5^+/−^) (Figure 1a). The rats had a 334 bp deletion resulting in C5 mRNA with premature stop codons, and nine G1 pups (C5^+/−^) were shipped to the University of Arkansas for Medical Sciences to establish the breeding colony. Breeding of the G1 C5^+/−^ rats produced a G2 generation of C5^−/−^ rats carrying the full deletion of C5 (100% knockout), which were compared with C5^+/−^ and C5^+/+^ littermates throughout these studies. Genomic DNA was extracted from G2 rat tail snips and was amplified via PCR using a C5 primer before visualizing the DNA bands on an agarose gel. As shown in Figure 1b, the C5^+/+^ DNA band appears at 789 bp and the C5^−/−^ DNA band appears at 334 bp, with C5^+/−^ rats displaying the bands for both alleles.

**FIGURE 2:**
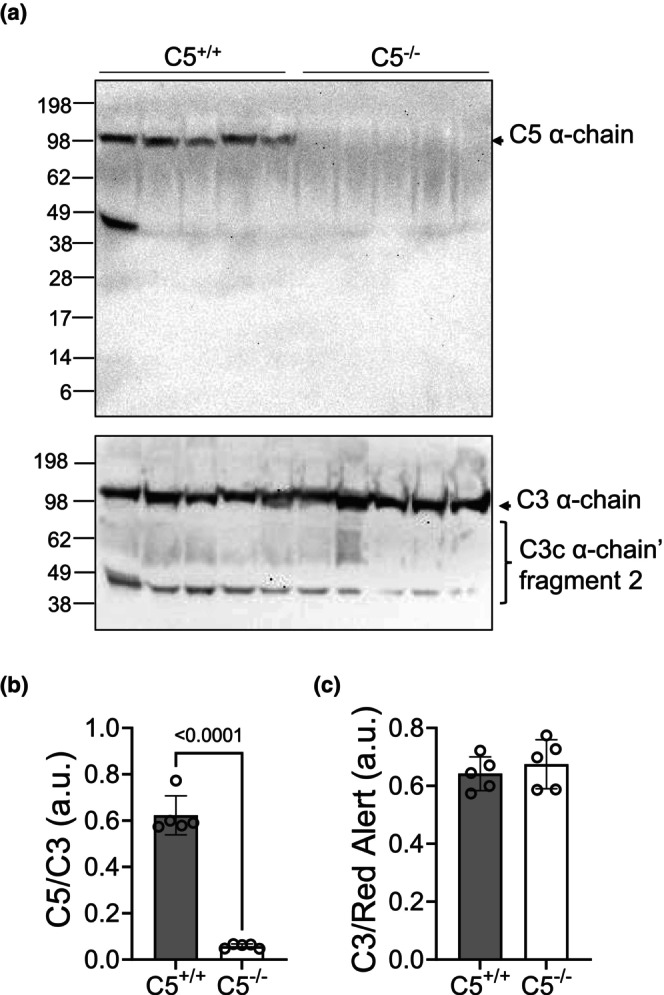
Serum validation of C5^−/−^. Blood samples from tail vein was collected from wild type (C5^+/+^) and homozygous (C5^−/−^) rats. (a) SDS‐PAGE Western blot of rat serum from C5^+/+^ and C5^−/−^ rats reveals an absence of C5 α‐chain (115 kDa) in C5^−/−^ rats (upper panel) but presence of C3, an earlier complement component, α‐chain and its degraded products in both genotypes. Molecular weight marker is indicated on the left. (b) Densitometry analysis of C5 relative to C3 reveals a significant decrease in the C5/C3 ratio in C5^−/−^ rats. (c) Densitometry analysis of C3 relative to total serum protein as quantified via Red Alert stain reveals no significant differences in C3 serum level between genotypes. Data are shown as the mean ± SD, *n* = 5. a.u., arbitrary unit.

**FIGURE 3:**
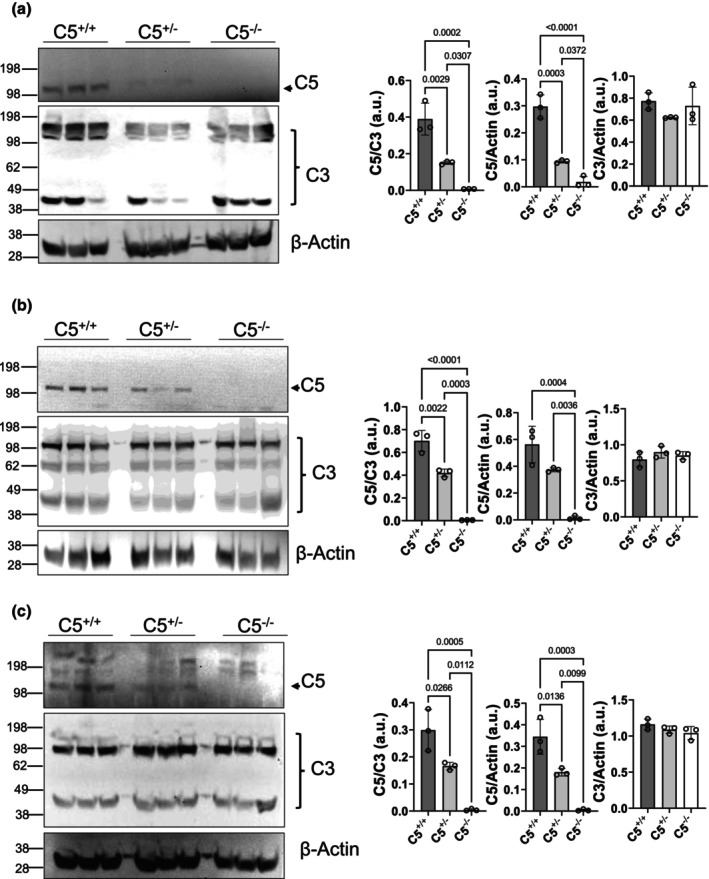
Tissue validation of global C5^−/−^. Frozen tissues (liver, kidney, and heart) from wild type (C5^+/+^), heterozygous (C5^+/−^), and homozygous (C5^−/−^) rats (*n* = 3/group) were homogenized and RIPA lysate was prepared. 20 μg RIPA lysate per rat per lane was resolved through a gradient gel (4%–12%) and Western blotting was performed using antibodies against C5 α‐chain, C3 α‐chain, and β‐actin. C3 α‐chain and β‐actin were used as controls. (a–c) SDS‐PAGE Western blot of liver tissue lysate (a), kidney tissue lysate (b), and heart tissue lysate (c) from male rats confirms the absence of C5 protein in the homozygous C5^−/−^ rats and reduced C5 protein in C5^+/−^ rats. Corresponding bar graphs show densitometry ratio of C5 and C3, C5 and β‐actin, and C3 and β‐actin. Data are shown as the mean ± SD, *n* = 3/group.

The vast majority of C5 is synthesized in the liver before being exported to the circulation, where it may be activated by earlier components of the complement cascade (Khanal, 2022). To validate the global deletion of C5, rat serum and liver tissue lysates were processed for Western blot and probed for C5. The C5 α‐chain (~115 kDa) can be visualized in the serum of C5^+/+^ rats but not C5^−/−^ littermates, indicating a lack of C5 circulating in the blood of the global C5^−/−^ rats (Figure 2a, upper panel, and Figure 2b). However, the C3 serum protein level was not affected by C5 deletion, indicating that earlier components of the complement cascade were preserved (Figure 2a, lower panel, and Figure 2c). To determine the liver‐specific deletion of C5 in C5^−/−^ rats, liver tissue, the primary location of C5 synthesis, was also processed for SDS‐PAGE and probed for C5 (Khanal, 2022). Results show that C5 depletion was achieved in C5^−/−^ rat whole liver tissue (Figure 3a). The liver is not the only potential source of C5 synthesis, as noted by prior reviews illuminating the role(s) of the intracellular complosome (Arbore et al., 2017; West & Kemper, 2023). Additionally, whole kidney and heart tissues were evaluated for the presence of C5. The kidney (Figure 3b) and heart lysates (Figure 3c) each displayed complete C5 depletion in knockout rats when compared to C5^+/+^ littermates. C5 depletion in not only the liver but also in the heart and kidney demonstrates that genomic deletion of C5 exon 3 results in the termination of C5 formation by the liver (eliminating circulating C5) as well as other tissues and cell types.

### 2.2 Augmentation of complement cascade gene expression in the kidney by C5 ^−/−^

**FIGURE 4:**
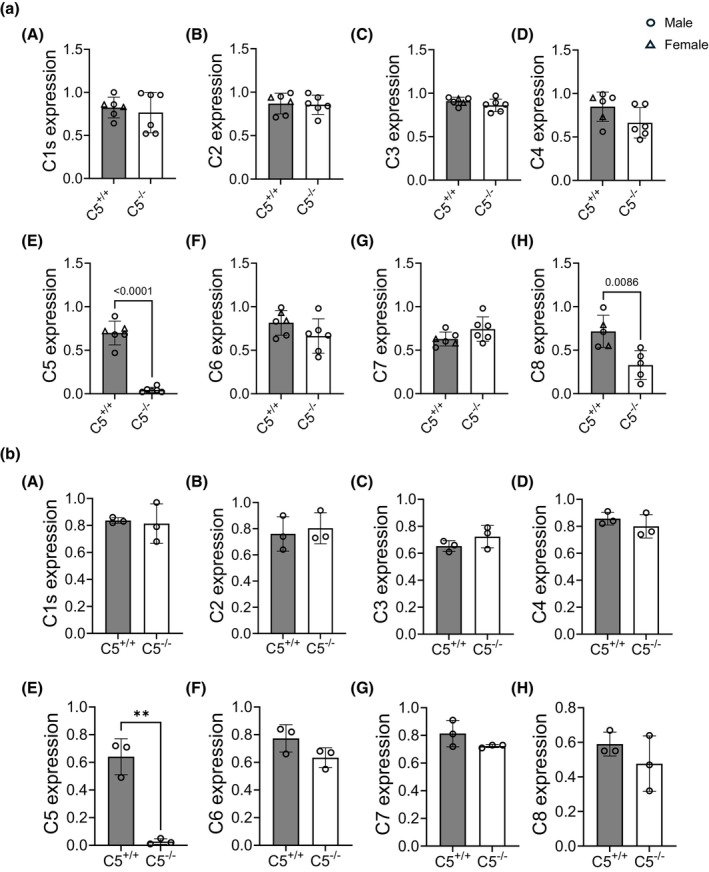
Gene expression of kidney and liver complosome components. Kidney or liver tissues derived from wild type (C5^+/+^) and homozygous (C5^−/−^) male and female rats were used to isolate RNA followed by cDNA synthesis. SYBR green PCR of cDNA was performed using primers of target genes (C1–C8). (a, A–H) Bar graphs showing relative mRNA expression (ΔΔC_t_) of C1, C2, C3, C4, C5, C6, C7, and C8 genes, respectively, in kidney tissues from C5^+/+^ and C5^−/−^ rats. Data are shown as the mean ± SD, *n* = 6/group. (b, A–H) Bar graphs showing relative mRNA expression (ΔΔC_t_) of C1, C2, C3, C4, C5, C6, C7, and C8 genes, respectively in liver tissues from C5^+/+^ and C5^−/−^ rats. Data are shown as the mean ± SD, *n* = 3/group.

The intracellular complosome has been implicated in a variety of cellular functions. Specifically, C3 has been previously reported to enter the nucleus in human B cells, strongly associating with the DNA and interacting with histone proteins to regulate transcription (Kremlitzka et al., 2019). It is possible that other complosome components have similar properties, and it is unknown how genomic deletion of a singular complement component could impact the transcription of other complement genes outside of the liver. To examine the effect of C5^−/−^ on intracellular complement component gene expression (C1–C8), RNA was isolated from kidney tissue and analyzed via RT‐PCR (Figure 4a A–H). Strikingly, complement C8 (but not other complement components) displayed significantly decreased gene expression in C5^−/−^ kidneys compared to C5^+/+^ (Figure 4a H). To confirm if this statistically significant finding was kidney‐specific, RNA was isolated from liver tissue and analyzed via RT‐PCR (Figure 4b). The liver is the primary source of circulating complement proteins. However, no differences in complement component (C1–C8) gene expression were observed in the liver (Figure 4b A–H), indicating that C5 gene deletion exerts a kidney‐specific effect on C8 gene expression.

### 2.3 C5 ^−/−^ rats show no physiologic differences in growth or tissue morphology from C5 ^+/+^ littermates

**FIGURE 5:**
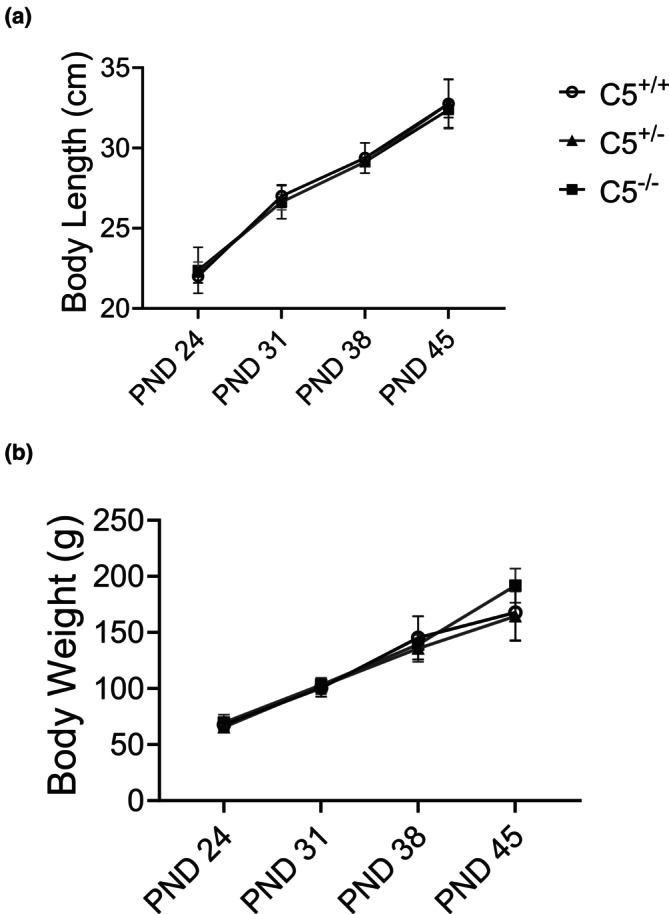
Animal growth for each genotype. Wild type (C5^+/+^), heterozygous (C5^+/−^), and homozygous (C5^−/−^) male rats were evaluated for animal growth patterns (a, b) Animal growth rate showing body length (a) and body weight (b) of male rats recorded weekly from postnatal day 24 to postnatal day 45. Data are shown as the mean ± SD, *n* = 4.

Transgenic rat models are an effective tool for investigating a wide variety of disease pathologies and are especially useful in dissecting the roles of genomically encoded proteins. However, transgenic animal models may display altered physiology or poor health compared to wild‐type counterparts due to unforeseen consequences of altering the genome (Filipiak et al., 2019; Reichardt & Fischer, 2016). As C5 is an important component of the innate immune system, there was a risk that C5^−/−^ rats could also experience poor health compared to C5^+/+^ littermates if the genomic deletion of C5 resulted in decreased innate immunity. To determine the overall health of C5^−/−^ rats, animal growth (in length and weight) was measured weekly postnatally. C5^−/−^ rats were not observed to have adverse health outcomes when compared to C5^+/+^ littermates, with no obvious signs of infection and similar lifespans. There were no significant differences in the body length (Figure 5a) and weight of C5^−/−^ pups (Figure 5b), even after 45 postnatal days. These results indicate that, at a baseline, the overall health and growth rate of C5^−/−^ rats do not differ from the C5^+/+^ group.

**FIGURE 6:**
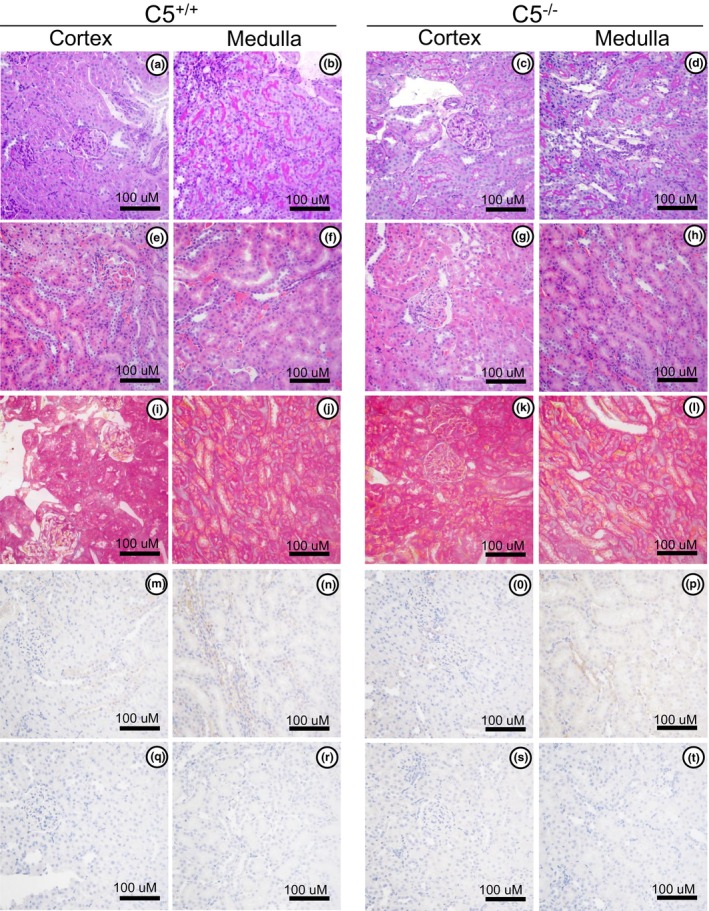
Characterization of C5^−/−^ kidney tissue morphology and injury markers. Kidney biopsies isolated from wild type (C5^+/+^) and homozygous (C5^−/−^) male and female rats were processed for formalin‐fixed paraffin‐embedded tissue blocks. Approximately 4–5 μM kidney sections (*n* = 4/group) were deparaffinized and hydrated. Morphological differences were evaluated via Periodic Acid‐Schiff stain (a–d) and Hematoxylin and Eosin stain (e–h). Renal fibrosis was evaluated using Masson's Trichrome staining (i–l). Rehydrated kidney sections (*n* = 4/group) were also employed for immunohistochemistry for kidney injury markers, KIM‐1 (m–p) and NGAL (q–t). All images were taken from randomized 40× fields in the kidney cortex or medulla (10 per animal; *n* = 4/group), and representative images were selected for each condition.

To determine the renal morphology of C5^−/−^ rats, kidney tissue cross‐sections were processed for histochemical evaluation. Compared to C5^+/+^ littermates, C5^−/−^ rats exhibited no differences in glomerular or tubular morphology in PAS (Figure 6a–d) and H&E (Figure 6e–h), suggesting a normal kidney architecture. Similarly, Masson's Trichrome staining shows similar levels of renal collagen between C5^−/−^ and C5^+/+^ rats (Figure 6i–l), indicating C5 deletion does not induce renal fibrosis. Furthermore, baseline staining for established kidney injury markers (KIM‐1 and NGAL) was analyzed via immunohistochemistry. As expected for healthy animals, baseline conditions did not produce deposition of KIM‐1 (Figure 6m–p) and NGAL (Figure 6q–t) in C5^−/−^ or C5^+/+^ rats.

### 2.4 C5 ^−/−^ rats exhibit no basal level changes in renal function, blood chemistry, or blood gases

**FIGURE 7:**
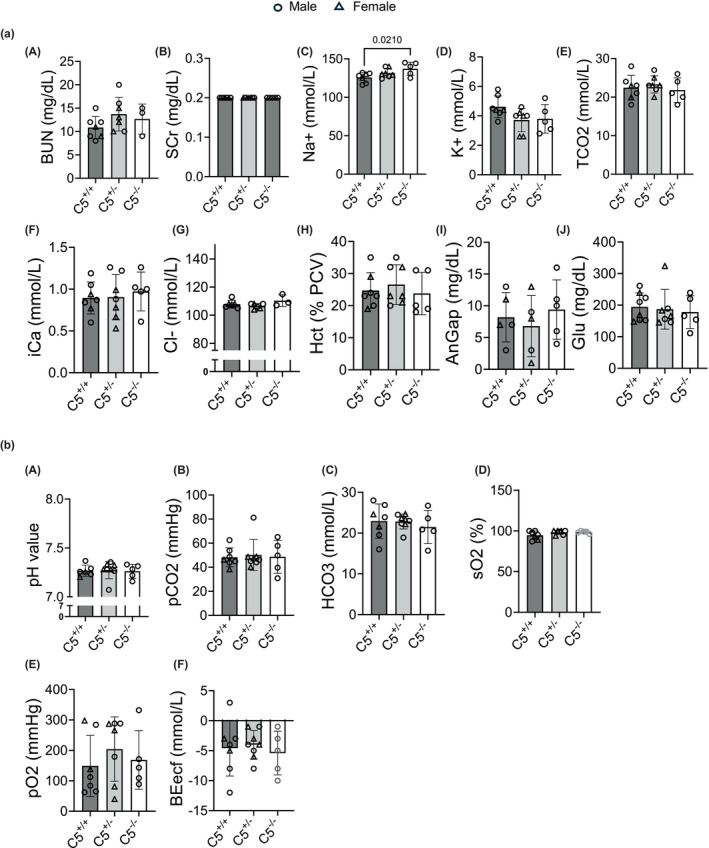
Characterization of blood chemistries and arterial blood gases for C5^−/−^ rats. Heparinized whole blood collected from male and female wild type (C5^+/+^), heterozygous (C5^+/−^), and homozygous (C5^−/−^) rats (*n* = 7/group) was immediately loaded into a cartridge for analysis via the VetScan i‐STAT system (Abaxis). (a) Bar graphs showing CHEM8+ cartridge‐derived blood chemistry values of blood urea nitrogen (BUN), serum creatinine (SCr), potassium ions (K+), total carbon dioxide (TCO_2_), ionized calcium (iCa), chloride ions (Cl−), hematocrit (Hct), anion gap (AnGap), or glucose (Glu) at baseline among the genotypes. *p < 0.05. (b) Bar graphs showing CG4+ cartridge‐derived arterial blood gas profile of pH, partial oxygen (pO_2_), partial carbon dioxide (pCO_2_), HCO_3_, base excess (BEecf), and oxygen saturation (sO_2_) revealed no differences between groups. Data are shown as the mean ± SD, *n* = 5–7.

The complement system, and specifically C5, remains a well‐known contributor to various kidney diseases and, in some cases, may perpetuate kidney tissue injury (Boudhabhay et al., 2020; Durigutto et al., 2017; Li et al., 2021). Therefore, it was relevant to evaluate the effect of C5^−/−^ alone on kidney function. Blood collected from C5^−/−^ rats was analyzed for blood chemistry (Figure 7a) and blood gas composition (Figure 7b) using a veterinary diagnostic analyzer (VetScan i‐STAT; Abaxis). Notably, there were no significant differences in blood urea nitrogen (BUN) (Figure 7a A) or serum creatinine (SCr) levels (Figure 7a B), both markers of kidney injury, between C5^−/−^, C5^+/−^, or C5^+/+^ groups, indicating an equivalent level of baseline kidney function. Similarly, no significant differences were observed in potassium (K), total carbon dioxide (TCO_2_), chloride (Cl), ionized calcium (iCa), hematocrit (Hct), anion gap (AnGap), or glucose (Glu) levels in the rat blood (Figure 7d–j). C5^−/−^ rats displayed a significant increase in blood Na^+^ levels compared to C5^+/+^ littermates (Figure 7c), but it is not clear if this statistical difference exerts a true physiologic impact, as both C5^+/+^ and C5^−/−^ Na^+^ levels fall within the previously published serum Na^+^ range for laboratory rats (Harkness et al., 2024; Suckow et al., 2020). These results suggest that C5^+/+^ and C5^−/−^ rats display no overt biological differences in blood chemistry.

In addition to blood chemistry, arterial blood gases were measured to determine the effect of C5^−/−^ on the animal's respiratory and metabolic status (Figure 7b). No significant differences were observed in arterial blood gas levels between groups, with the following parameters measured: pH, partial carbon dioxide (pCO_2_), partial oxygen (pO_2_), bicarbonate (HCO_3_), base excess (BEecf), and oxygen saturation (sO_2_) (Figure 7b A–F). Notably, C5^−/−^ rats displayed no signs of metabolic acidosis, another indication that C5^−/−^ rats do not display compromised kidney function at baseline.

### 2.5 Global C5 ^−/−^ does not affect blood immune cell composition at baseline

**FIGURE 8:**
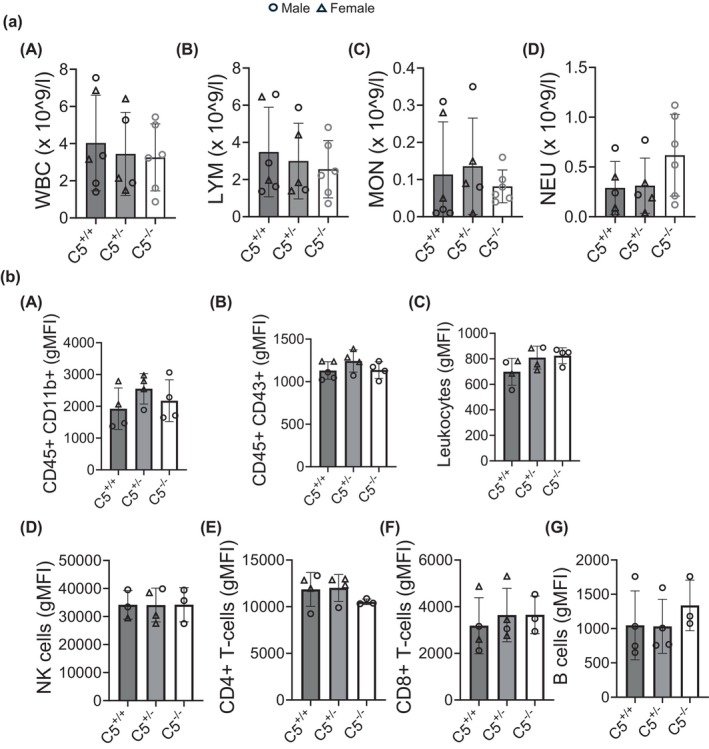
Baseline circulating immune cell composition in C5^+/+^ versus C5^−/−^. Heparinized whole blood collected from wild type (C5^+/+^), heterozygous (C5^+/−^) and homozygous (C5^−/−^) male and female rats (*n* = 4–6/group) was processed for total blood count and characterization of immune cell composition via flowcytometry. (a) Whole blood was processed for total blood counting using VetScan HM5 (Abaxis). Total count (×10^9^/L) of white blood cells (WBCs, A), lymphocytes (LYM, B), monocytes (MON, C), and neutrophils (NEU, D). (b) Rat whole blood‐derived WBCs after RBC lysis were stained for immune cell surface markers as indicated followed by flowcytometry analysis. Bar graphs showing quantification of CD45+CD11b+ (A), CD45+CD43+ (B), CD45+ leukocytes (C), NK cells (D), CD4+ T‐cells (E), CD8+ T‐cells (F), or B cells (G). Data are shown as the mean ± SD, *n* = 3–4 (some samples were excluded from data analysis due to an early processing error).

C5 deletion results in diminished innate immunity by virtue of an incomplete complement cascade, possibly impacting the composition of immune cells as a compensatory mechanism. A cleaved product of C5, the C5a anaphylatoxin, is a strong chemotactic factor that interacts with its receptor on the surfaces of various immune cell subtypes (Ghosh & Rana, 2023). The loss of C5a‐C5aR signaling in C5^−/−^ rats, therefore, has the potential to impact the overall immune cell composition. C5aR1, for example, has previously reported expression on cells of myeloid and lymphoid origin (Soruri et al., 2003). In addition to well‐established roles in granulocyte and macrophage chemotaxis, C5a‐C5aR signaling has been reported to modulate effector T‐cell function as well as germinal center B cell chemotaxis (Kupp et al., 1991; Lalli et al., 2008). While C5aR expression in NK cells remains unclear (Fusakio et al., 2011; Min et al., 2014), C5a‐C5aR signaling may impact NK cell activation via a dendritic cell‐dependent mechanism (Qing et al., 2012). Therefore, we sought to elucidate the impact of C5 gene deletion on immune cell count and composition in the blood. To assess the levels of immune cell subtypes in C5^−/−^ rats, collected whole blood was processed for total blood cell count and flow cytometry. Total blood counts revealed no significant differences in the cell number of WBCs, lymphocytes, monocytes, and neutrophils among C5^+/+^, C5^+/−^, and C5^−/−^ rats at baseline (Figure 8a A–D). Immune cell lineages were assessed via flow cytometry using markers for leukocytes (CD45+), CD45+CD11b+ (myeloid lineage), CD45+CD43+ (monocyte lineage), T cells (CD4+ and CD8+), natural killer (NK) cells (CD161a+), and B cells (CD45R+) (Figure 8b A–G). The gating strategy, outlined in Figure S1, was adapted from a prior 9‐color flow cytometric method to characterize major leukocyte populations in the rat (Barnett‐Vanes et al., 2016). Contrary to our hypothesis, C5^−/−^ rats displayed no statistically significant differences in circulating immune cell composition compared to C5^+/+^ littermates (Figure 8b A–G), suggesting that the ablation of C5 has little impact on immune cell compartments.

**FIGURE 9:**
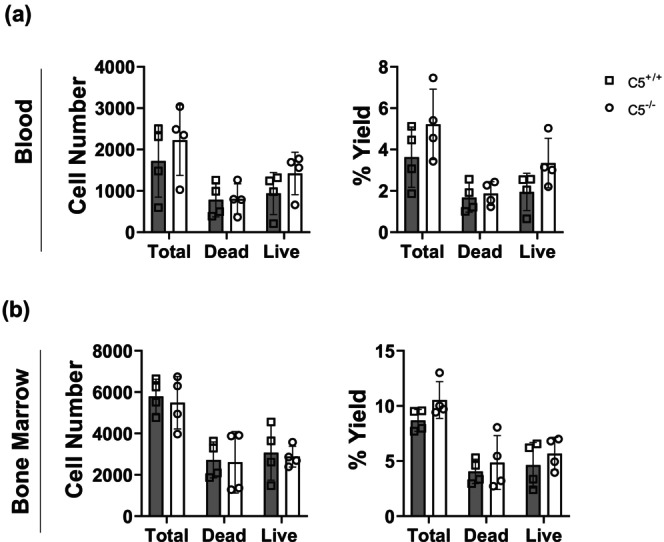
Status of circulating neutrophils in C5^+/+^ versus C5^−/−^ at basal conditions. Whole blood samples and bone marrow were isolated from wild type (C5^+/+^) and homozygous (C5^−/−^) rats and processed for RBC lysis. Resulting while blood cells (WBCs) were employed forcell counting via trypan blue hemocytometry. Cells were then stained for FACS analysis of neutrophil surface markers utilizing the antibodies shown in Table 2. Bar graphs showing neutrophil cell number (left panel) or percentage (%) yield (right panel) of neutrophils in blood (a) and bone marrow (b). Data are shown as the mean ± SD, *n* = 4.

After C5 is activated, its cleaved product C5a is known to bind to its G‐protein receptor C5aR1 to produce a strong chemotactic signal in neutrophils and other innate immune cells (Miyabe et al., 2017; Sadik et al., 2018). Therefore, the composition of neutrophils in the circulating blood and bone marrow was compared between C5^−/−^ and C5^+/+^ littermates (Figure 9). Both circulating neutrophils (Figure 9a) and the neutrophil population in the bone marrow (Figure 9b) are unaffected by C5 deletion at baseline.

### 2.6 C5 ^−/−^ improves kidney function and ameliorates necrosis post‐IRI

**FIGURE 10:**
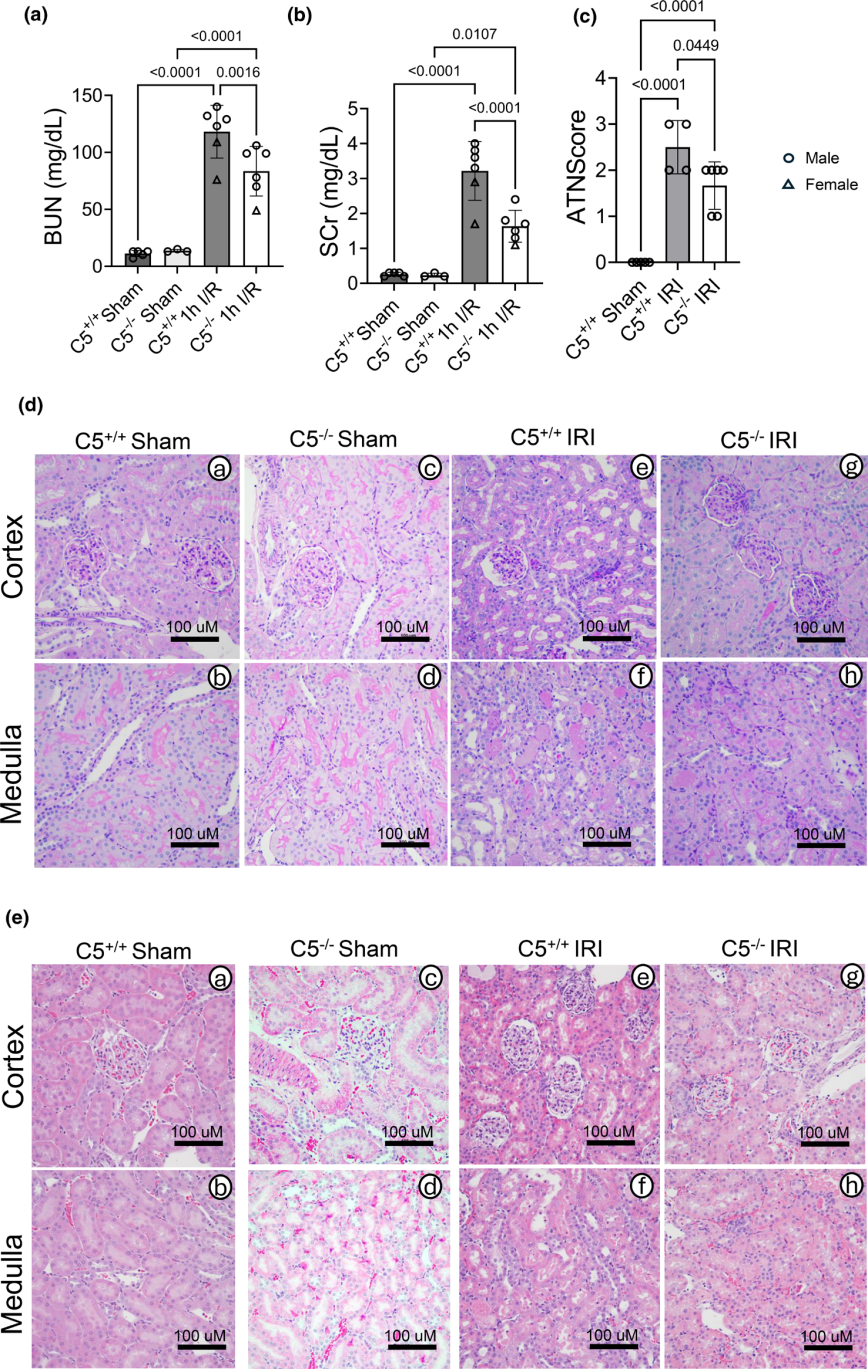
C5^−/−^ improves kidney function and mitigates kidney tissue injury after ischemia and reperfusion (IRI). Wild type (C5^+/+^) and homozygous (C5^−/−^) rats (male and female) were employed for bilateral renal ischemia (30 min) plus reperfusion (1 day) injury (IRI). Sham surgery (right nephrectomy) served as a control for both genotypes. Blood and kidneys were isolated post‐surgery and employed for kidney function/injury assays (*n* = 3–6/group). (a, b) Bar graphs showing kidney function test post‐surgery. Blood urea nitrogen (BUN) (a) and serum creatinine (SCr) (b) were quantified via the VetScan i‐STAT system. Data are shown as the mean ± SD, *n* = 4–5. (c–e) Formalin‐fixed kidney sections isolated from rats 1 day post‐surgery were stained with PAS (d) or H&E (e). (c) Bar graph showing acute tubular necrosis (ATN) score as quantified by a blinded pathologist. Data are shown as the mean ± SD, *n* = 4–5. (d) Representative micrographs (*n* = 5/group) from cortex and medulla stained with PAS. (e) Representative micrographs (*n* = 5/group) from cortex and medulla stained with H&E.

Complement activation is a hallmark of IRI, and the cleaved components of C5 play pivotal roles as a part of the terminal complement pathway. We characterized the kidney function and tissue morphology in C5^+/+^ and C5^−/−^ rats post‐IRI to investigate the role of C5 in acute kidney tissue injury in rats. As expected, the BUN and SCr levels in C5^−/−^ rats were significantly decreased compared to C5^+/+^ post‐injury, suggesting that C5^−/−^ protects kidney function (Figure 10a,b). As expected, the acute tubular necrosis (ATN) score (scoring was conducted by a blinded, licensed pathologist) in C5^+/+^ rats was significantly increased after IRI when compared to C5^+/+^ sham rats (Figure 10c). Furthermore, C5^−/−^ post‐IRI rats displayed significantly decreased ATN score compared to C5^−/−^ sham and C5^+/+^ post‐IRI rats (Figure 10c), indicating the tissue morphology was preserved. In representative images from C5^+/+^ and C5^−/−^ rats post‐IRI, C5^−/−^ conserved the renal tubular morphology and ameliorated necrosis as visualized via PAS (Figure 10d A–H) and H&E staining (Figure 10e A–H).

### 2.7 C5 ^−/−^ ameliorates complement deposition and apoptosis post‐IRI

**FIGURE 11:**
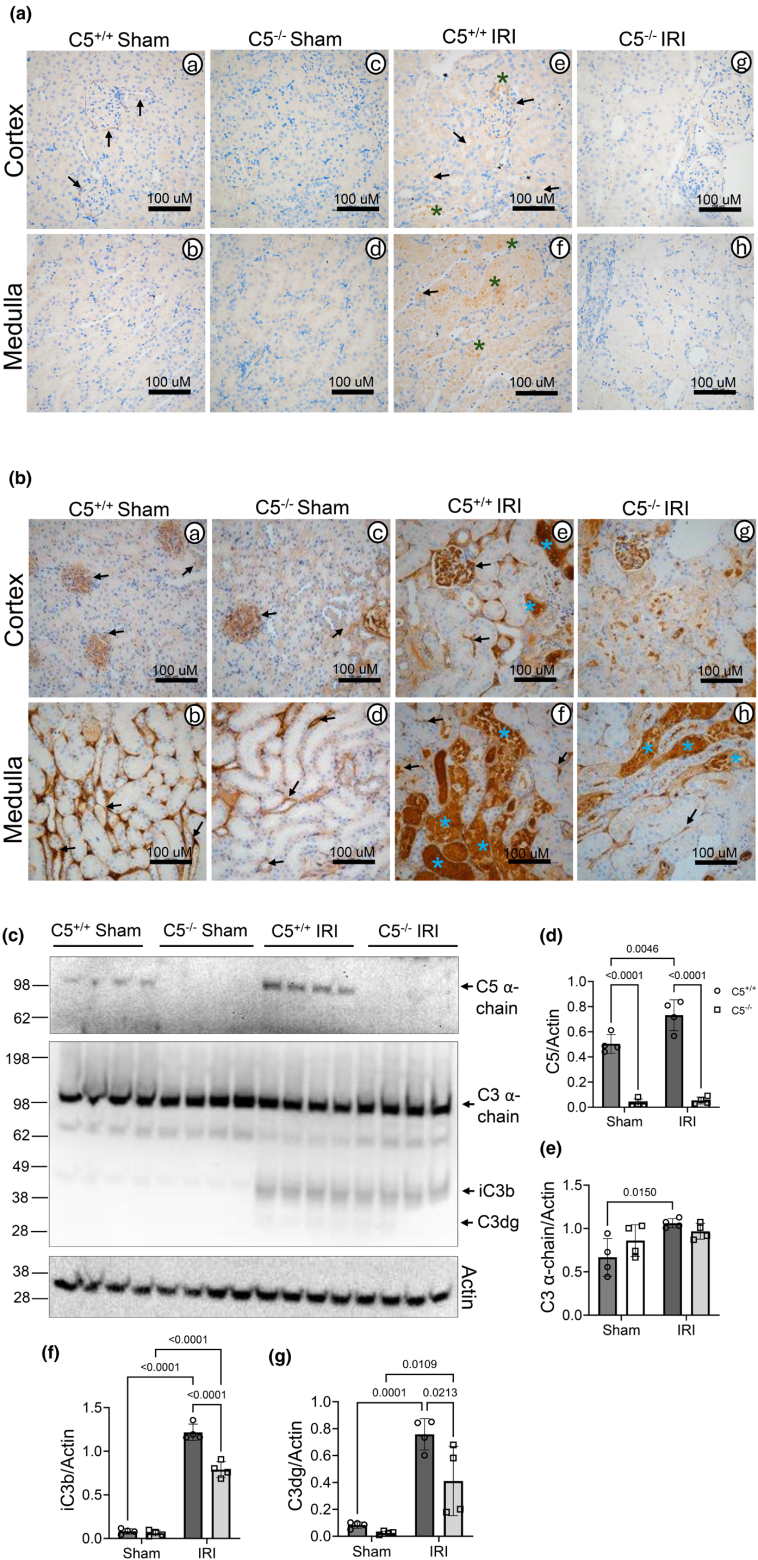
C5−/− ameliorates complement deposition post‐IRI. Wild type (C5^+/+^) and homozygous (C5^−/−^) rats (male and female) were employed for bilateral renal ischemia (30 min) plus reperfusion (1 day) injury (IRI). Sham surgery (right nephrectomy) served as control for both genotypes. Kidneys were isolated post‐surgery and processed for formalin‐fixed paraffin‐embedded sections or biochemical analysis. (a, b) Immunohistochemistry assay for deposition of complement components C5 (a) and C3 (b). Arrows indicate C5/C3 deposition in glomerular/tubular/peritubular regions and asterisks indicate intracellular C5/C3 deposition. (c–g) Whole kidney tissue lysates from rats 1 day post‐surgery were processed for SDS‐PAGE Western blotting followed by densitometry analysis. (c) Representative Western blot image of C3 and C5. β‐actin was used as control. (d) Bar graph showing densitometry analysis of C5/ β‐actin ratio. Data are shown as the mean ± SD, *n* = 4/group. (e) Bar graph showing densitometry analysis of C3α‐chain/β‐actin ratio. Data are shown as the mean ± SEM, *n* = 4/group. *p < 0.05. (f) Bar graph showing densitometry analysis of iC3b/ β‐actin ratio. Data are shown as the mean ± SD, *n* = 4/group. (g) Bar graph showing densitometry analysis of C3dg/β‐Actin ratio. Data are shown as the mean ± SD, *n* = 4/group.

We determined the impact of C5 gene deletion on the deposition of complement components C5 and C3 in the kidney tissue post‐IRI (Figure 11). Immunohistochemical analysis of formalin‐fixed kidney tissue sections revealed a basal level of C5 confined in the glomerular capsule and peri‐tubules in C5^+/+^ sham rats (Figure 11a A and B, indicated by arrows). As expected, C5 protein was absent in C5^−/−^ sham rats (Figure 11a C and D). Notably, following IRI, C5 protein was increased in glomeruli, renal tubules, and peri‐tubules (Figure 11a E and F, indicated by arrows), including intracellular C5 deposition (Figure 11a E and F, indicated by asterisks) in the C5^+/+^ model. Similar to sham, the C5^−/−^ IRI model shows null C5 protein levels in kidneys (Figure 11a G and H).

Immunohistochemical analysis of kidney sections revealed a basal presence of C3 protein in glomerular capillaries and peritubular compartments of C5^+/+^ (Figure 11b A and B, indicated by arrows) and C5^−/−^ sham rats (Figure 11b C and D, indicated by arrows). Similar to the C5 component, IRI increased C3 protein deposition in glomerular capillaries, renal tubules, and peri‐tubules of both C5^+/+^ (Figure 11b E and F, indicated by arrows) and C5^−/−^ rats (Figure 11b G and H, indicated by arrows) when compared to both C5^+/+^ and C5^−/−^ sham rats. However, C3 deposition was remarkably reduced in C5^−/−^ rats, particularly in the renal medulla (Figure 11b E–H). Notably, a profound increase in intracellular deposition of C3 was evident in C5^+/+^ kidneys after IRI when compared to sham (Figure 11b E and F, indicated by asterisks), and C5^−/−^ IRI model exhibited reduced intracellular C3 deposition when compared to C5^+/+^ IRI kidneys (Figure 11b E–H, indicated by asterisks), suggesting less tubular damage in C5^−/−^ post‐IRI rats.

To further confirm the immunohistochemical analysis, the total protein levels of C5 and C3 were assessed via SDS‐PAGE Western blot in whole kidney lysate (Figure 11c–g). As expected, C5^−/−^ sham and IRI groups displayed the complete attenuation of C5 protein compared to C5^+/+^ sham and IRI groups. Notably, the C5^+/+^ IRI model displayed an increase in the C5 protein level compared to the C5^+/+^ sham control (Figure 11d). All of the groups displayed the presence of the C3 α‐chain (115 kDa), which is enzymatically cleaved from the C3 β‐chain to enable the formation of C3a and C3b (Bokisch et al., 1975). The C5^+/+^ IRI model displayed a significant increase in the C3 α‐chain protein level compared to sham controls (Figure 11e), while there were no significant differences detected in the C3 α‐chain level between C5^−/−^ IRI and sham groups (Figure 11e). C3 is activated when C3 is cleaved by the C3 convertase into C3a and C3b; the C3b component amplifies the complement immune response by forming the C5 convertase, or alternatively, acting as a key opsonin mediating various innate and adaptive immunity functions. Furthermore, the degradation of C3b results in the formation of many degraded products, including iC3b, C3c, and C3dg (Ricklin et al., 2016). While C3c is quickly released into the circulation, both iC3b and C3dg may remain bound to the cell surface to exert further signaling functions (Lo et al., 2021; Ricklin et al., 2016). Western blots show that the C5^+/+^ and C5^−/−^ IRI groups each displayed an increase in C3 cleaved/degraded products (Figure 11c) compared to sham controls. This suggests the occurrence of C3 activation following IRI in both groups. Densitometry evaluations showed C5^−/−^ IRI group displayed decreased levels of iC3b and C3dg levels compared to C5^+/+^ post‐IRI rats, further confirming reduced levels of C3b or blunted activation of C3.

**FIGURE 12:**
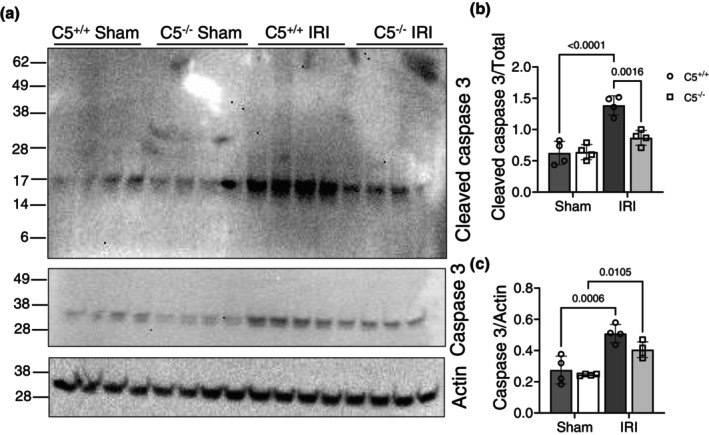
C5 gene deletion modulates apoptosis post‐IRI. Wild type (C5^+/+^) and homozygous (C5^−/−^) rats (male and female) were employed for bilateral renal ischemia (30 min) plus reperfusion (1 day) injury (IRI). Sham surgery (right nephrectomy) served as control for both genotypes. Kidneys were isolated post‐surgery and processed for formalin‐fixed paraffin‐embedded sections or biochemical analysis. (a–c) Whole kidney tissue lysates (RIPA) from rats 1 day post‐surgery were processed for SDS‐PAGE Western blotting (caspase‐3) followed by densitometry analysis. (a) Representative Western blot image of cleaved (upper panel) and total caspase 3 (middle panel). β‐actin was used as control (lower panel). (b) Bar graph showing densitometry analysis of cleaved/total caspase 3 ratio. Data are shown as the mean ± SD, *n* = 4/group. (c) Bar graph showing densitometry analysis of total caspase 3/β‐actin ratio. Data are shown as the mean ± SD, *n* = 4/group.

Tubular cell death by necrosis and apoptosis is a major mechanism that is associated with renal IRI. Whereas apoptosis minimizes the release of cell contents that can trigger inflammation, the acute tubular necrosis observed during IRI is not as well controlled. A decrease in overall complement deposition suggests a decrease in MAC formation, a mechanism of cell lysis mentioned previously (Vlaicu et al., 2019). However, this does not exclude the potential contributions of other types of cell death, such as apoptosis. We analyzed kidney tissue lysates via Western blotting to determine the expression of caspase 3, an executioner of apoptosis (Nydam et al., 2018) (Figure 12). As expected, both C5^+/+^ and C5^−/−^ rats displayed basal levels of total and cleaved caspase 3 in sham kidneys (Figure 12a–c). Similarly, we observed a significant increase in the protein levels of cleaved and total caspase 3 (Figure 12a–c) in C5^+/+^ rat kidneys following IRI. Similar to the C5^+/+^ group, IRI significantly increased protein levels of total caspase 3 in C5^−/−^ kidneys when compared to C5^−/−^ sham groups (Figure 12a,c). Strikingly, C5^−/−^ resulted in a significant decrease in the cleaved caspase 3 protein level post‐IRI compared to C5^+/+^ littermates, suggesting that C5 gene deletion blunted cleavage of caspase 3 and mitigated apoptosis in kidney tissue post‐IRI.

### 2.8 Blood immune cell composition and tissue infiltration are modulated by C5 ^−/−^ post‐IRI

**FIGURE 13:**
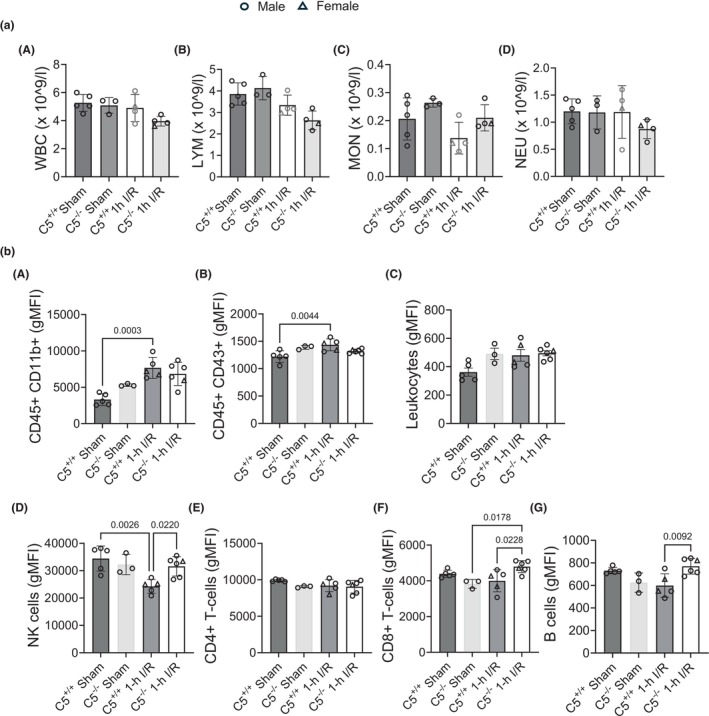
Alterations in circulating immune cell composition were observed post‐IRI in C5^+/+^ and C5^−/−^. Wild type (C5^+/+^) and homozygous (C5^−/−^) rats (male and female) were employed for bilateral renal ischemia (30 min) plus reperfusion (1 day) injury (IRI). Sham surgery (right nephrectomy) served as control for both genotypes. Blood was isolated post surgery and processed for hematology (total blood count) and flowcytometry analyses. (a) Heparinized whole blood was processed for total blood counting via VetScan HM5 (Abaxis). (A–D) Bar graphs showing total count (×10^9^/L) of white blood cells (WBCs, A), lymphocytes (LYM, B), monocytes (MON, C), and neutrophils (NEU, D). (b) Characterization of immune cell composition via flowcytometry. Rat whole blood‐derived WBCs (after RBC lysis) were stained for immune cell surface markers as indicated and evaluated by flowcytometry analysis. Bar graphs showing quantification of circulating CD45+CD11b+ (A), CD45+CD43+ (B), CD45+ leukocytes (C), NK cells (D), CD4+ T‐cells (E), CD8+ T‐cells (F), or B cells (G) in blood. Data are shown as the mean ± SD, *n* = 3–6/group.

Total blood counts for the IRI model and sham controls were obtained from whole rat blood (Figure 13a A–D). No significant differences in total blood counts were observed for total WBCs (Figure 13a A), lymphocytes (Figure 13a B), monocytes (Figure 13a C), or neutrophils (Figure 13a D). To further assess changes in circulating immune cell composition in response to IRI, white blood cells from C5^+/+^ and C5^−/−^ rats post‐IRI were analyzed by flow cytometry (Figure 13b A–G). C5^+/+^ and C5^−/−^ sham groups were used as controls. As in baseline (Figure 8b), sham surgery did not modulate circulating immune cells between the genotypes (Figure 13b). In C5^+/+^ rats, post‐IRI displayed increased circulating CD45+CD11b+ (myeloid lineage) and CD45+CD43+ (monocyte lineage) cells (Figure 13b A and B), as well as CD8+ T‐cells (Figure 13b F) compared to the C5^+/+^ sham control. These data suggest that IRI spurs the production of myeloid cells and monocytes, as well as the chemotaxis of CD8+ T‐cells. C5^−/−^ rats post‐IRI followed a similar trend from C5^−/−^ sham controls, although only CD8+ T‐cells were significantly increased (Figure 13b F). No significant differences were observed in CD45+ leukocytes or CD4+ T‐cells for any experimental group (Figure 13b C and E). In contrast, CD45R+ B cells were significantly elevated in C5^−/−^ rats post‐IRI compared to the C5^+/+^ IRI group (Figure 13b G). Furthermore, circulating CD161a+ NK cells showed striking differences between groups/genotypes. While circulating NK cells were significantly decreased in C5^+/+^ post‐IRI compared to the C5^+/+^ sham control, the C5^−/−^ IRI group did not display this decrease and maintained sham levels of circulating NK cells post‐IRI. This resulted in significantly different numbers of circulating NK cells in C5^−/−^ rats post‐IRI compared to the C5^+/+^ IRI group (Figure 13b D). This observation reinforces a role for C5 and C5a‐C5aR signaling in NK cells.

**FIGURE 14:**
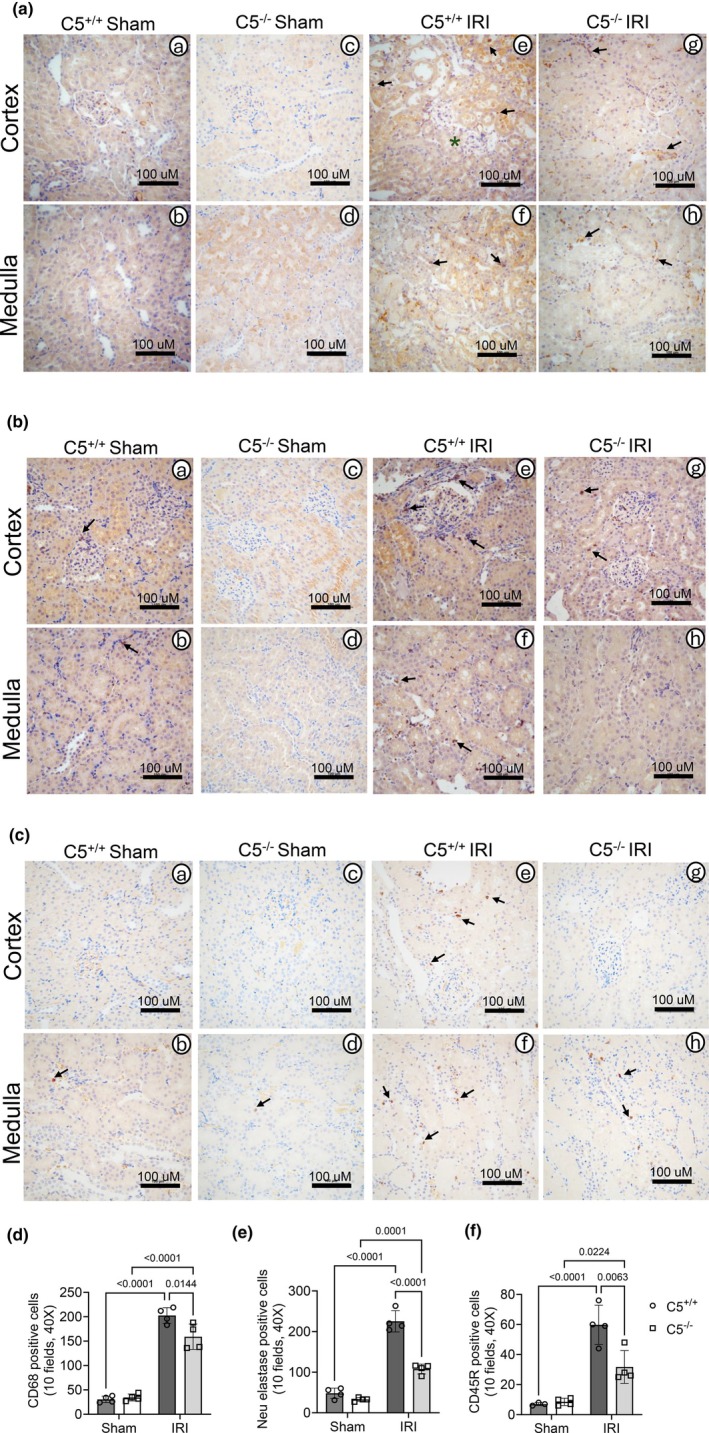
Immune cell infiltration in kidney tissue post‐IRI. Wild type (C5^+/+^) and homozygous (C5^−/−^) rats (male and female) were employed for bilateral renal ischemia (30 min) plus reperfusion (1 day) injury (IRI). Sham surgery (right nephrectomy) served as a control for both genotypes. Kidneys were isolated post‐surgery and processed for formalin‐fixed paraffin‐embedded sections. (a–f) Immunohistochemistry assay for infiltration of immune cells and quantitative analysis. Representative micrographs of CD68+ cells (a), neutrophil elastase (b), and CD45R+ B cells (c) after immunohistochemistry assay. Arrows in a–c indicate respective positive cells in kidneys. (d–f) Bar graphs showing quantification of positive cells per 10 fields (40×) for CD68+ cell count (d), neutrophil elastase+ cell count (e), and CD45R+ cell count (f). Data are shown as the mean ± SD, *n* = 4/group.

Chemotactic signaling by complement anaphylatoxins, including C5a, is known to promote immune cell infiltration to sites of tissue injury (Khanal, 2022; Peng et al., 2012). Because C5 gene deletion modulated circulating immune cell composition, we sought to determine the impact of C5 gene deletion on immune cell infiltration in kidneys post‐IRI. Formalin‐fixed kidney sections were analyzed via immunohistochemistry for macrophages, neutrophils, and B cells (Figure 14). Immunohistochemistry revealed sporadic and insignificant numbers of CD68+, neutrophil elastase+, and B cells in rat kidneys in C5^+/+^ and C5^−/−^ sham groups (Figure 14a–f). Both C5^+/+^ and C5^−/−^ IRI groups displayed increased infiltration of CD68+ macrophages (Figure 14a A–H and Figure 14d), neutrophils (Figure 14b A–H and Figure 14e), and B cells (Figure 14c A–H and Figure 14f) compared to respective sham controls. Interestingly, macrophage, neutrophil, and B cell infiltration in kidneys was significantly attenuated in the C5^−/−^ post‐IRI group (Figure 14a G and H; Figure 14d) compared to littermate C5^+/+^ rats (Figure 14a–f). Together, these observations confirm a role for C5a in immune cell chemotactic signaling to injured tissue.

## 3 DISCUSSION

We have presented above the robust characterization of a novel, global C5^−/−^ model in the rat. In summary, while C5^−/−^ rats displayed few physiologic differences from C5^+/+^, deletion of C5 conferred a protective effect post‐renal IRI similar to murine models of C5 deletion (Boor et al., 2007; Zhou et al., 2000). Overall, deletion of C5 exon 3 resulted in no ill effects in the rodents at baseline, indicating that C5 may be a nonessential gene in healthy conditions. C5^−/−^ rats displayed similar growth patterns, blood immune composition, and kidney physiology to their C5^+/+^ counterparts. The gene expression of other kidney complosome components was largely unaffected by C5^−/−^ except, interestingly, C8 gene expression. However, the application of an acute kidney injury model (IRI) revealed robust differences between the C5^+/+^ and C5^−/−^ groups. C5^−/−^ improved kidney function and ameliorated tissue necrosis and apoptosis post‐IRI, and C5^−/−^ rats displayed evidence of an altered immune response to injury. These results taken together suggest that C5 activation plays a significant role in the development of renal ischemia‐reperfusion injury in a rat model and contributes to inflammation and necroptosis processes.

Our results confirm previous murine studies conducted by Zhou et al., which concluded that C3, C5, and C6 deficiencies exerted a protective effect in renal IRI (Zhou et al., 2000). In this study, C5 deficiency was achieved in mice via a spontaneous mutation of the complement component, and the kidneys were harvested at 72 h post‐injury, differing from our IRI model, which utilizes CRISPR/Cas9‐mediated exon 3 deletion of C5 combined with 24 h post‐IRI. The protective effect of C6 deficiency observed by Zhou et al. suggests the importance of C5a‐C5aR signaling in renal IRI, as C6‐deficient mice can generate C5a but cannot form MAC. Further, murine C5^−/−^ models have demonstrated a role for C5 in the development of renal tubulointerstitial fibrosis, as genetic C5 deficiency was protective in a model of unilateral ureteral ligation (Boor et al., 2007).

The “complosome,” or intracellular complement components that are synthesized locally, has been previously implicated in kidney failure due to ischemic injury (Farrar et al., 2006). We investigated the impact of C5 deletion on the gene expression of other complosome components in the kidney. Some complement proteins, such as C3, have been reported to associate with histone proteins in the nucleus to augment transcription (Kremlitzka et al., 2019). However, the ability of C5 to take on such a role has yet to be studied. Therefore, it was striking that C5^−/−^ resulted in decreased C8 mRNA expression in the kidney, but not in the liver. C8 is somewhat unique among complement proteins, as it consists of three subunits that are encoded by entirely different genes. The C8γ subunit gene (in the rat) is made up of 7 exons on chromosome 3 (3p13), and this is the same chromosome where the C5 gene is encoded (3p11). In humans, the C5 and C8γ genes are located on chromosome 9 at 9q33.2 and 9q34.3, respectively. Though it is unlikely that C5 exon deletion interfered with C8γ transcription directly, further studies should address possible co‐regulatory mechanisms between C5 and C8 gene expression in the kidney.

Here we report, for the first time, the attenuation of IRI‐mediated apoptosis via C5 gene deletion. Interestingly, our study demonstrated that C5 gene deletion blunts IRI‐mediated upstream complement cascade and caspase 3 activation, suggesting that complement pathway activation induces the apoptotic pathway. In previous studies, both the sublytic deposition of MAC (Liu et al., 2013) and the C5a anaphylatoxin (Flierl et al., 2008; Tsai et al., 2019) have been linked to apoptosis induction. While the lytic MAC complex formed via activation of the complement cascade shares some similarities with apoptosis (namely, nucleolar changes), cell lysis via MAC more closely corresponds to necrosis (loss of cell volume) (Vlaicu et al., 2019). Further, anti‐C5a treatment in a rodent model of sepsis completely inhibited the activation of caspase‐3, ‐6, and ‐9 (Guo et al., 2000). Future studies should address the crosstalk between C5/C5a and modes of apoptosis with respect to caspase 3 as well as the other extrinsic and intrinsic pathways.

We observed differences in circulating immune cell composition between C5^+/+^ and C5^−/−^ post‐IRI, but no significant differences were present at baseline. This suggests that the activation of the complement cascade and the cleavage of C5 into its subunits is key to the immune response to IRI. As briefly discussed, C5a plays a significant role in inflammatory signaling when it binds to C5aR1, its G‐protein receptor (Ghosh & Rana, 2023; Huber‐Lang et al., 2001). Most famously known as a chemotactic signal, C5a‐C5aR1 binding on the surface of immune cells drives immune cell infiltration to injured tissue (Ghosh & Rana, 2023; Huber‐Lang et al., 2001; van den Bos et al., 2020). A mechanistic study with C5aR1^−/−^ mice further demonstrated crosstalk between C5a‐C5aR1 and the induction of NOD‐like receptor pyrin domain‐containing protein 3 (NLRP3) inflammasome, the complex responsible for cell pyroptosis and pro‐IL‐1β processing (Haggadone et al., 2016). In this way, C5a‐C5aR signaling assists in the regulation of multiple tightly controlled processes intracellularly, including the immune response. In this study, circulating levels of CD8+ T‐cells, B cells, and NK cells were altered due to C5^−/−^ after IRI. The C5^−/−^ IRI group additionally displayed decreased renal infiltration of macrophages, neutrophils, and B cells, indicating the role of C5 in enhancing tissue infiltration of major immune cells.

CD8+ T‐cells have been reported to plasma membrane C5a receptors, and C5aR antagonism has additionally been reported to regulate T‐cell proliferation (Cravedi et al., 2013; Nataf et al., 1999). In cancer, there is a growing recognition of C5aR as an immune checkpoint receptor, and C5aR deletion enhanced T‐cell antitumor activity via IL‐10 (Wang et al., 2019). Depletion of CD8+ T‐cells abolished the effect (Vadrevu et al., 2014). Similarly, B cells have been shown to respond chemotactically to C5a via C5aR interactions, and rC5a has been reported to increase the polarization and migration of human B cells (Kupp et al., 1991; Ottonello et al., 1999). As circulating CD8+ T‐cells and B cells were both increased in C5^−/−^ rats compared to C5^+/+^ post‐IRI, and B cells also demonstrated decreased tissue infiltration, it is possible that the loss of C5a impaired these chemotactic/migratory processes in T‐cells and B cells. Future studies are warranted to demonstrate the specific role of C5a and/or C5b in modulating immune cells during renal IRI.

Strikingly, the IRI‐mediated loss of circulating NK cells in C5^+/+^ rats was reversed in C5^−/−^. It is not clear whether IRI increases NK cell infiltration in rat kidneys and this effect is modulated by C5 deletion. Despite our efforts, the available commercial antibodies against NK cells did not appear to work for immunohistochemistry using kidney sections. Future studies need to address this issue in kidneys post‐IRI with respect to C5 proficiency/deficiency. The expression of C5aR receptors on NK cells is controversial, as some studies report CD56+ NK cell expression of C5aR (Lubbers et al., 2017; Min et al., 2014) and others reported expression only under septic conditions (Fusakio et al., 2011). Still others have observed that C5 activates dendritic cells and NK cells via an indirect mechanism of TGF‐β production, and neither cell type expressed C5aR (Qing et al., 2012). Further study is needed to elucidate the conditions in which the C5aR may be expressed and the nature of C5a‐C5aR signaling in NK cells.

The complement system, specifically C5, has been implicated in a wide range of pathologies—including Alzheimer's, retinopathies, diabetes, autoimmune disorders, and classical diseases of “complement” such as atypical hemolytic uremic syndrome (aHUS) (Ardissino et al., 2021; Ishii et al., 2021; Krance et al., 2021; Li et al., 2021; Torres et al., 2021; Trakkides et al., 2019). In the cardiovascular system, C5 has been reported to exacerbate atherosclerosis, and C5aR1 antagonism was linked to the amelioration of atherosclerotic disease in murine models (Liu et al., 2023). Recently, C5a‐C5aR1 signaling was more specifically implicated in activating a pathway of endoplasmic reticulum (ER) stress that promoted vascular calcification (Liu et al., 2023). It has also been well‐documented that C5a‐C5aR1 binding promotes innate immune cell inflammasome activation (Arbore et al., 2016; Haggadone et al., 2016) and IL‐1β production, both key steps in a pro‐inflammatory amplification loop that drives atherogenesis (Kalbitz et al., 2016; Libby, 2017; Triantafilou et al., 2013). Thus, the value of animal models that target the role of C5 in cardiovascular disease research is clear. In such studies, rats have a significant advantage over their murine counterparts due to their 10‐fold greater myocardial mass, wider arterial lumens, and increased blood volume.

Kidney health reflects a key facet of cardiovascular research and is the focus of our laboratory. In the kidney, C5 blockade has revolutionized the treatment of aHUS (Fakhouri et al., 2023; Mauch et al., 2023) and paroxysmal nocturnal hemoglobinuria (PNH) (Stern & Connell, 2019), both diseases where MAC‐mediated endothelial cell damage represents a crucial therapeutic target. Furthermore, complement therapies have reduced the need for corticosteroids in both ANCA‐associated glomerulonephritis (Trivioli & Vaglio, 2020) and IgA nephropathy (Maillard et al., 2015). The role of C5 is also being studied in C3 glomerulopathy, a rare disease of the kidney in which acquired autoantibodies target the C3 or C5 convertases, dysregulating complement activation (Smith et al., 2019). Global genomic deletion of C5 offers a new angle from which to approach the study of complement‐mediated diseases in the kidney while eliminating the potential off‐target effects of pharmacologic inhibitors.

To summarize, we report the generation and characterization of a novel, CRISPR/Cas9‐mediated C5^−/−^ in the rat. Specifically, C5 exon 3 deletion resulted in no adverse impact on rodent growth, blood chemistry, arterial blood gases, or immune composition at baseline. In the kidney, healthy C5^−/−^ tissue displayed no significant morphological or fibrotic changes from C5^+/+^ counterparts as demonstrated via PAS, H&E, and Masson's Trichrome staining. We also observed a decrease in C8 gene expression in the kidney mediated by C5^−/−^, and future studies should address the driving mechanisms of complosome transcription. After ischemia‐reperfusion injury, C5^−/−^ rats displayed improved kidney function and decreased tissue necrosis compared to C5^+/+^, indicating C5 activation potentiates adverse outcomes in renal IRI. C5^−/−^ deletion also altered the whole blood immune cell composition post‐IRI consistent with the loss of chemotactic factor C5a. Most importantly, as aberrant complement activation (either circulating or via the intracellular complosome) continues to grow as a field spanning multiple pathologies and tissue types, the need for robust animal models targeting the role(s) of complement components has increased. This novel CRISPR/Cas9‐mediated C5^−/−^ rat may offer distinct advantages over murine models and thus can overcome difficulties in pharmacologic targeting.

## 4 METHODS

### 4.1 Generation of a novel C5 knockout rat model

CRISPR/Cas9‐mediated knockout of C5 in Lewis rats was performed by the University of Michigan Transgenic Core. Briefly, CRISPR reagents microinjected into fertilized rat eggs were used to induce double‐strand breaks in C5‐201 exon 3 (located on chromosome 3 in the rat), and base pair removal was carried out via nonhomologous end‐joining repair using a previously described protocol (Filipiak & Saunders, 2006). The resulting mRNA contained premature termination codons. Tail snip biopsies were collected, and genomic DNA was prepared using the DNEasy DNA kit (Qiagen, #69504). DNA samples were analyzed via PCR for deletion products (Table 1). Pups that were positive for the deletion allele (G0) were mated to wild‐type Lewis female rats and produced 9 G1 pups heterozygous for the deletion allele, which were shipped to the University of Arkansas for Medical Sciences.

**TABLE 1:** Primers.

Primer	Source	Sequence
C1s	RTPrimers	Forward 5′‐TATGTCGGGTCCACACTTCT‐3′; Reverse 5′‐GTCCGTGTATTCAGGTCGTC‐3′
C2	RTPrimers	Forward 5′‐CCATGTGGAGGAGGTGTTAG‐3′; Reverse 5′‐GTGGCTGGACATTTTCACTC‐3′
C3	RTPrimers	Forward 5′‐GTACTTGGGAGACGTGGATG‐3′; Reverse 5′‐ATGAGGGTGTTCTTGTTGGA‐3′
C4	RTPrimers	Forward 5′‐AGACAGCATGAAGCTCCAAC‐3′; Reverse 5′‐AAGACCTTGCTCATGTCGAG‐3′
C5	RTPrimers	Forward 5′‐GCTTTTATTCCACCCAGGAT‐3′; Reverse 5′‐TCCCCTTTGTGTTTGTAGGA‐3′
C6	RTPrimers	Forward 5′‐CTCACTGGTCCAGCTGTTCT‐3′; Reverse 5′‐ACTGTCTGGTCTCCTGCTTG‐3′
C7	RTPrimers	Forward 5′‐GGGAGAAACTGCAGAACTCA‐3′; Reverse 5′‐CTTGCCAACGAGAGCATAAT‐3′
C8	RTPrimers	Forward 5′‐GCTGAGGGAGAACAAGTCAA‐3′; Reverse 5′‐AGTCTCCTCCCTGCTGATCT‐3′

### 4.2 Animal care and colony maintenance

Male and female G1 Lewis rats were obtained from the University of Michigan Transgenic Core and maintained in a colony for the following studies. Heterozygous (C5^+/−^) males and females were housed in monogamous breeding pairs through the female's ovulation cycle, and then males were removed from the cage once pregnancy was confirmed. Males were reintroduced after the litters were weaned at ~3 weeks post‐birth for further breeding. The rats were maintained with a 12 h day/night cycle, housed at physiological temperatures, and were fed standard chow (Lab Diet, #3002906‐704) and water ad libitum. All of the animal use protocols were approved by an Institutional Animal Care and Use Committee (IACUC) at the University of Arkansas for Medical Sciences. Animal experiments were performed according to the criteria outlined by the National Institutes of Health (NIH).

### 4.3 Genotype analysis

Genomic DNA was extracted from tail snip biopsies (3–5 mm) obtained postweaning using the Phire Direct PCR Master Mix kit (Thermofisher Scientific, #F170S). The C5 primer sequence routinely used to detect C5 gene deletion was as stated in Table 1 (sourced from RealTimePrimers.com), and a 334 bp deletion product was observed. PCR conditions were as follows: 98°C for 5 min initial denaturation, 40 cycles of 98°C for 5 s and 70°C for 30 s for annealing and extension. PCR products were visualized on a 1.5% agarose gel prepared with 1× Tris‐Borate‐EDTA (TBE) buffer (Invitrogen, #15581‐028) and ethidium bromide (Invitrogen, #15585‐011). DNA bands were visualized using the iBright 1500 (Invitrogen, #A44241).

### 4.4 Animal surgery

#### 4.4.1 Ischemia‐reperfusion injury procedure

Male and female Lewis rats (8–10 weeks) were utilized for these studies, undergoing a model of renal ischemia‐reperfusion injury (IRI). Rats were first anesthetized via isoflurane (5% induction and 2% maintenance anesthesia) using an ISOTEC vaporizer. A vertical incision was made from the xyphoid process to an area superior to the symphysis pubis, exposing the interior abdomen. Both renal pedicles were clamped for a total time of 1 h. After this period of ischemia, a right nephrectomy was performed, and the left kidney clamp was removed to allow reperfusion of the left kidney (IRI). The muscular layer of the abdominal incision was sutured, and the outer layer of skin was stapled. The rats were then allowed to recover from anesthesia and returned to the animal facility. After 1‐day post‐surgery (IRI), the rats were re‐anesthetized and the left kidney, liver, heart, lungs, spleen, bone marrow, and blood were collected. Animals were euthanized via exsanguination as approved in the animal use protocol. Blood was processed for serum, plasma, and blood composition analysis. Tissues were processed for (1) Western blot analysis, (2) histochemical analysis, and (3) isolation of RNA.

#### 4.4.2 Sham procedure

Sham surgeries were performed similarly except for the IRI. Briefly, rats were anesthetized via isoflurane, and a vertical incision was made to expose the interior abdomen. A right nephrectomy was performed, and the right kidney was preserved as a healthy control (untreated). After the wound closure, the rats were returned to the animal facility for 1‐day post‐surgery (sham) and were re‐anesthetized for the collection of the left kidney, liver, heart, lungs, spleen, bone marrow, and blood and further processed as described under Ischemia‐Reperfusion Injury.

### 4.5 Hematology

Blood collected from rats was deposited into Eppendorf tubes (serum and blood chemistry/blood gas analysis) or EDTA tubes (total blood counts). Fresh whole blood was analyzed using CG4+ (Abaxis, #03P85‐25) and CHEM8+ cartridges (Abaxis, #09P31‐26) and the VetScan 1 i‐STAT (Abaxis, #04P75‐03) to obtain blood gas and blood chemistry values, respectively. Collected blood was analyzed using the VetScan HM5 (Abaxis, #790‐0000) for complete blood counts or centrifuged at 350 × *g* for 10 min to collect serum.

### 4.6 Flow cytometry

Blood samples in sodium heparin were lysed in 1× ACK Lysis Buffer (Gibco, #A1049201), and cell counting was performed for blood and bone marrow via trypan blue hemocytometry. Cells were stained with MACS buffer (PBS + 0.1% BSA, 2 mM EDTA, pH 8.0) containing live‐dead fixability dye (Biolegend, #423102) and Fc‐blocked (BD, #550270) at 4°C for 10 min to decrease nonspecific binding. The cells were incubated with surface markers using antibodies as depicted in Table 2. After incubation at 4°C for 30 min, cells were washed with MACS buffer and fixed in 2% paraformaldehyde for 20 min. Fixed cells were analyzed using the BD LSRFortessa (BD Biosciences) with appropriate compensation controls. Data were analyzed using FlowJo software version 10.8.1.

**TABLE 2:** Antibodies.

Antibody	Source	Application	Dilution	Catalog No. #
CD45‐AF700	Biolegend	FACS	1:200	202218
CD3‐APC	Biolegend	FACS	1:200	201414
CD4‐FITC	Biolegend	FACS	1:200	201505
CD8a‐BV650	BD	FACS	1:200	563234
CD45R‐BV711	BD	FACS	1:200	563892
CD161a‐PE	BD	FACS	1:200	555009
CD43‐PEcy7	Biolegend	FACS	1:200	202816
HIS48‐FITC	BD	FACS	1:200	554907
CD11b‐BV605	BD	FACS	1:200	563015
C5	Proteintech	WB; IHC	1:1000 WB; 1:2000 IHC	22492‐1‐AP
C3	Abcam	WB; IHC	1:1000 WB; 1:8000 IHC	ab200999
Cleaved Caspase‐3	CST	WB	1:1000	9661
Caspase‐3	CST	WB	1:1000	9662
Β‐Actin	Sigma	WB	1:1000	A5441
KIM‐1	LSBio	IHC	1:2000	LS‐C312791
NGAL	LSBio	IHC	1:2000	LS‐C37211
CD68	Thermofisher	IHC	1:2000	PA5‐78996
Neutrophil Elastase	CST	IHC	1:400	440305
CD45R	Life Tech	IHC	1:800	14‐0460‐82
Peroxidase Goat Anti‐Mouse IgG	Jackson ImmunoResearch	WB	1:30,000	115‐035‐166
Peroxidase Goat Anti‐Rabbit IgG	Jackson ImmunoResearch	WB	1:30,000	111‐035‐144

### 4.7 SDS‐PAGE and Western blot analysis

Kidney extracts from whole kidney homogenates or frozen kidney tissue powder were prepared using complete radioimmunoprecipitation assay (RIPA) lysis buffer (Pierce, #89900), which contained 1.2 mM Na_3_VO_4_, 2.5 mM NaF, 1 mM DTT (BioRad, #161‐0611), 1 mM PMSF (Millipore, #532332), and protease inhibitor cocktail (Pierce, #1860932) as previously described (Parajuli et al., 2011). Centrifugation was performed after lysis at 16,000 × *g* for 20 min at 4°C, and the supernatant was preserved. Protein concentrations were determined using the BCA Protein Assay kit (Pierce, #23225). Kidney extracts were separated via SDS‐PAGE and transferred to a PVDF membrane. For analysis of serum samples, 2 μL of rat serum was boiled in sample loading buffer before separation via SDS‐PAGE. After transfer, membranes were incubated for 5 min at room temperature with 1× Red Alert Stain (Millipore, #71078) to visualize protein bands. The stain was lifted using quick washes with double‐distilled H_2_O, and membranes were blocked (5% dry nonfat milk in TBS‐T) and then incubated with primary antibodies, as shown in Table 2. Beta‐actin was used as a loading control. Probed membranes were washed with TBS‐T and incubated with horseradish peroxidase (HRP)‐conjugated secondary antibodies, as shown in Table 2. Imaging for chemiluminescence was performed using the iBright 1500 (Invitrogen, #A44241) and SuperSignal West Pico PLUS Chemiluminescent Substrate (Thermofisher Scientific, #34580), and densitometry was performed using AlphaEase FC software (Alpha Innotech).

### 4.8 Quantitative real‐time PCR

An RNEasy kit (Qiagen, #74136) was used to isolate total RNA from kidney tissue, and Superscript III (Invitrogen, #18080‐051) was used for reverse transcription of mRNA. RT‐PCR was carried out using the PowerUp SYBR Green Master Mix (Applied Biosystems, #A25742), and the PCR was run for 44 cycles with the following conditions: 95°C for 30 s, 58°C for 30 s, and 72°C for 1 min. Amplification of target genes was normalized to Actin amplification and to the levels of control using the ΔΔC_t_ method (Parajuli et al., 2010). The primer sequences (sourced from RealTimePrimers.com) were as depicted in Table 1.

### 4.9 Histology and immunohistochemistry

Paraffin blocks were prepared from formalin‐fixed tissue, and kidney sections were mounted on glass slides (Fisher Scientific, #12‐544‐3) in two cross‐sections, each 4–5 μm in thickness (Lo et al., 2021). Cross‐sections were deparaffinized through xylene and a series of graded ethanol washes, and the sections were further processed for Periodic Acid‐Schiff (PAS) (Parajuli et al., 2011), Hematoxylin and Eosin (H&E), and Masson's Trichrome staining. For immunohistochemistry, kidney cross‐sections underwent antigen retrieval via heating in sodium citrate buffer (pH 6.0), and sections were quenched with BLOXALL™ Endogenous Peroxidase and Alkaline Phosphatase Blocking Solution (Vector, #SP‐600) (Lo et al., 2021). Sections were blocked with 2.5% normal goat serum and incubated in primary antibodies as depicted in Table 2. Immunoreactivity was detected with ImmPRESS™ Reagent Anti‐Rabbit IgG (for rabbit primary antibodies; Vector, #MP‐7451) or Anti‐Mouse IgG (for mouse primary antibodies; Vector, #MP‐7423) and ImmPACT™ DAB Peroxidase Substrate (Vector, #SK‐4105). Counterstaining was carried out using Mayer's Hematoxylin (Electron Microscopy Science, #26043), and bluing was performed (0.125% ammonia hydroxide). The slides were dehydrated and covered with Cytoseal‐60 (Electron Microscopy Science, #18006) before being mounted. Histochemical images were taken using a Nikon Eclipse E800 microscope and Nikon Elements software. A blinded licensed pathologist carried out histopathological evaluation and acute tubular necrosis (ATN) scoring.

### 4.10 Statistical analysis

Statistical analysis was performed using GraphPad Prism (Version 10), and the data are presented as mean ± SD. The Shapiro–Wilk normality test was performed to verify the normal distribution of the data. We performed one‐way or two‐way ANOVA and Tukey's post hoc (alpha = 0.05) test for multiple comparisons with Bonferroni's correction when the normal distribution assumptions were met. For the skewed data, the Mann–Whitney *U* test was performed to compare the difference between the two groups. Statistical significance was defined as a *p* value <0.05. Graphs were generated using GraphPad Prism.

## AUTHOR CONTRIBUTIONS

N.P. was involved in conceptualization, resources, supervision, and funding acquisition. M.M., D.B., A.S., N.J., S.L., and G.V. were involved in methodology and analysis. M.M. was involved in investigation. N.P. and M.M. were involved in data curation and writing—original draft preparation. N.P. and A.S. were involved in writing—review and editing. N.G., M.M., and N.P. were involved in visualization. All authors have read and agreed to the published version of the manuscript.

## FUNDING INFORMATION

NIH NIDDK (R01 DK123264) and UAMS Barton Pilot award (17‐DN‐08).

## CONFLICT OF INTEREST STATEMENT

The authors declared no competing interests.

## ETHICS STATEMENT

Animal studies were approved by an Institutional Animal Care and Use Committee (IACUC) at the University of Arkansas for Medical Sciences (Approval No. IPROTO202400000031).

## Supporting information


Figure S1.

## Data Availability

All data associated with this study is present in the paper. Data will be made available upon reasonable request.
