# Supplementary material for: Post‐ischemia and reperfusion kidney injury is mitigated in a novel complement 5 knockout rat
Source: Physiol Rep. 2025 Nov 5;13(21):e70576. doi: 10.14814/phy2.70576 (PMC12586936; doi:10.14814/phy2.70576)
Supplement: Supplementary file 1 — Figure S1. [file PHY2-13-e70576-s001.zip › caption.docx]

**Supp. Fig. 1. Gating strategy for blood immune cell analysis.** Whole blood samples were lysed with 1x ACK Lysis Buffer and cells were stained with live-dead fixability dye. Each sample was then stained utilizing two panels. Panel A: CD45-AF700, CD45R-BV711, CD161a-PE, CD3-APC, CD4-FITC, and CD8a-BV650. Panel B: CD45-AF700, CD11b-BV605, and CD43-PEcy7. All antibodies are as shown in Table 2. Samples were then fixed and analyzed using the BD LSRFFortessa (BD Biosciences) along with fluorescence minus one (FMO) controls. (A) Gating strategy: First we applied a viability gate (LIVEDEAD) to isolate living cells and a size gate to select single cells. These cells were gated for CD45+ and these were split into CD45R+ (B cells), CD161a+, and CD3+ cells. CD3+ cells were gated into CD4+CD8- (CD4+ T-cells) and CD8+CD4- (CD8+ T-cells) populations, respectively. Cells highly positive for CD161a and low side scatter were taken as NK cells. (B) Gating strategy: Viability gate, singlets, and CD45+ gating were all performed as in the top panel, then split into CD45+CD11b+ and CD45+CD43+ populations.
