# Supplementary figures and images for: Post‐ischemia and reperfusion kidney injury is mitigated in a novel complement 5 knockout rat
Source: Physiol Rep. 2025 Nov 5;13(21):e70576. doi: 10.14814/phy2.70576 (PMC12586936; doi:10.14814/phy2.70576)

Supplementary Figure 1

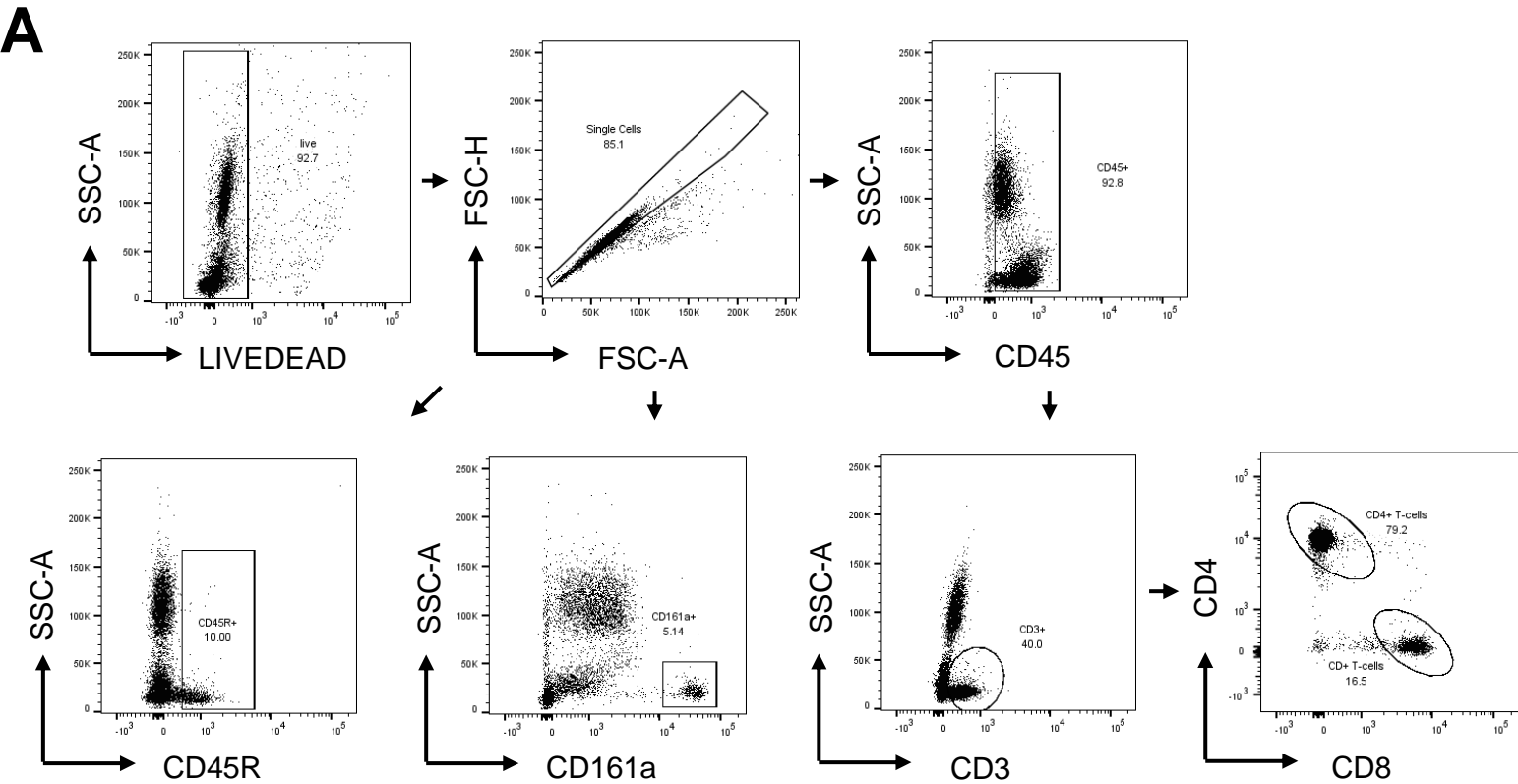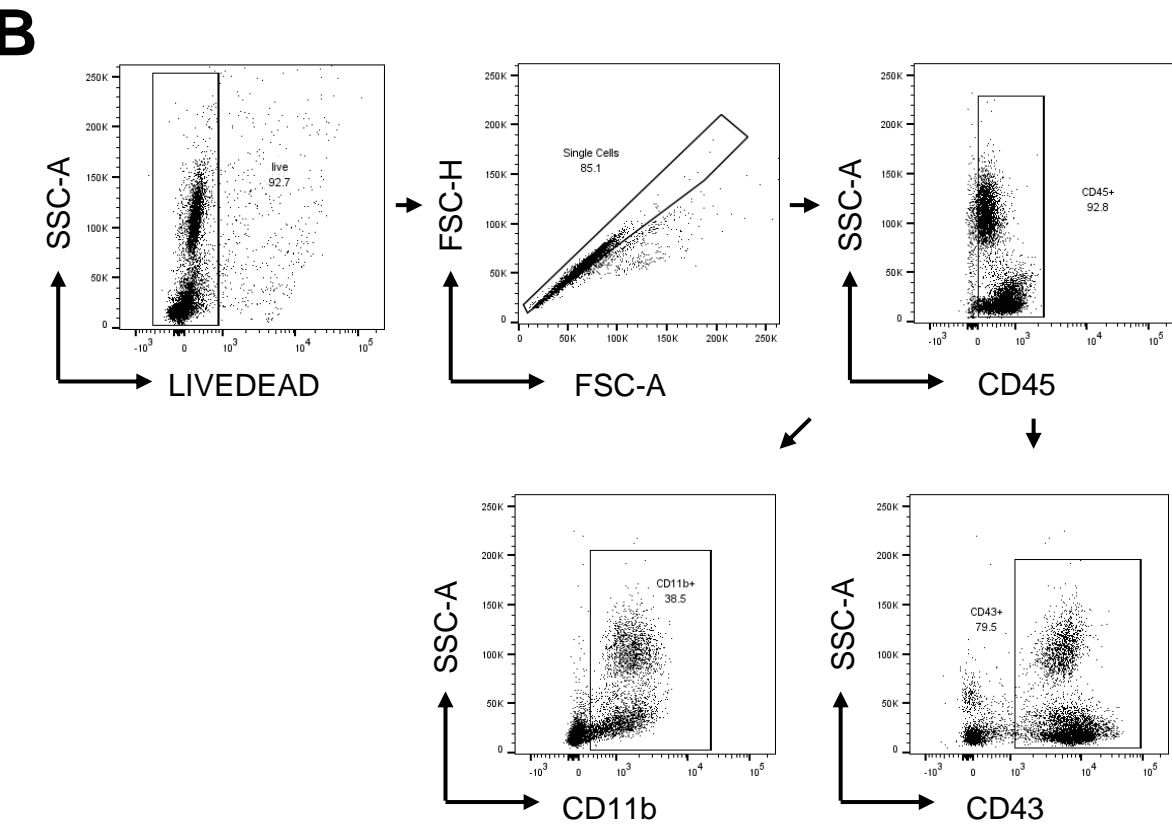

Supplement: Supplementary file 1 — Figure S1. [file PHY2-13-e70576-s001.zip › phy270576-sup-0001-FigureS1.pdf]
